# Increased Tumor Ascorbate is Associated with Extended Disease-Free Survival and Decreased Hypoxia-Inducible Factor-1 Activation in Human Colorectal Cancer

**DOI:** 10.3389/fonc.2014.00010

**Published:** 2014-02-04

**Authors:** Caroline Kuiper, Gabi U. Dachs, Delwyn Munn, Margaret J. Currie, Bridget A. Robinson, John F. Pearson, Margreet C. M. Vissers

**Affiliations:** ^1^Centre for Free Radical Research, Department of Pathology and Biomedical Science, University of Otago Christchurch, Christchurch, New Zealand; ^2^Mackenzie Cancer Research Group, Department of Pathology and Biomedical Science, University of Otago Christchurch, Christchurch, New Zealand; ^3^Canterbury Regional Cancer and Blood Service, Canterbury District Health Board, Christchurch Hospital, Christchurch, New Zealand; ^4^Biostatistics and Computational Biology Unit, Department of Public Health and General Practice, University of Otago Christchurch, Christchurch, New Zealand

**Keywords:** colorectal cancer, ascorbate, hypoxia-inducible factor, hydroxylation, VEGF, BNIP3

## Abstract

Ascorbate is a co-factor for the hydroxylases that regulate the transcription factor hypoxia-inducible factor (HIF)-1, which provides cancer cells with a metabolic and survival advantage in the hypoxic environment of solid tumors. However, whether ascorbate affects tumor development is a highly debated issue. We aimed to determine whether tumor ascorbate was associated with HIF-1 activation and patient disease-free survival. In this study, we undertook a retrospective observational analysis of tissue-banked tumor and paired normal tissue from 49 colorectal cancer patients, measuring ascorbate levels, HIF-1α and its downstream gene products BNIP3, and vascular endothelial cell growth factor (VEGF). Patient survival was monitored for the first 6 years after surgery. We found that ascorbate levels were lower in tumor tissue compared to normal tissue (*p* < 0.001) but overall levels varied considerably. HIF-1α, VEGF, and BNIP3 were elevated in tumor samples (*p* < 0.01). There was an inverse relationship between tumor ascorbate content and HIF-1 pathway activation (*p* = 0.002) and tumor size (*p* = 0.018). Higher tumor ascorbate content was associated with significantly improved disease-free survival in the first 6 years after surgery (*p* = 0.006), with 141–1,094 additional disease-free days. This was independent of tumor grade and stage. Survival advantage was associated with the amount of ascorbate in the tumor, but not with the amount in adjacent normal tissue. Our results demonstrate that higher tumor ascorbate content is associated with decreased HIF-1 activation, most likely due to the co-factor activity of ascorbate for the regulatory HIF hydroxylases. Our findings support the need for future studies to determine whether raising tumor ascorbate is possible with clinical intervention and whether this results in modification of hydroxylase-dependent pathways in the tumor.

## Introduction

Hypoxia-inducible factor (HIF)-1 is a transcription factor that up-regulates the expression of hundreds of genes that enable the successful adaptation of solid tumors to the metabolic challenges of a hypoxic microenvironment (1, 2). Increased HIF-1 has been linked to poor patient prognosis (3–6) and is implicated in radio- and chemo-resistance (4, 7) and metastasis (8, 9). Consequently, there is great interest in potential inhibitors of HIF-1 for use in cancer therapy (10, 11).

Hypoxia-inducible factor-1 activity is controlled by post-translational hydroxylation of the α subunit: hydroxylation of prolines 402 and 564 by prolyl-hydroxylases (PHDs) targets HIF-1α for rapid proteasomal degradation, and hydroxylation of asparagine 803 by factor inhibiting HIF (FIH) prevents HIF-1 co-activation with p300 (12). Thus, optimal PHD and FIH activity directly inhibits HIF-1 transcriptional activity. The HIF hydroxylases are members of the 2-oxoglutarate-dependent dioxygenase family (13) that also includes the collagen prolyl-4-hydroxylase (C-P4H) and jumonji histone demethylases (JmjC family) (14). These enzymes have a non-heme iron in the catalytic site and they require oxygen and 2-oxoglutarate as substrates (15) and ascorbate as a co-factor (12, 13). The absence of ascorbate compromises HIF-hydroxylase activity and leads to increased activation of HIF-1 in both cancer and primary cells *in vitro* (16–18).

The amount of intracellular ascorbate required for optimal HIF-hydroxylase activity is reflected in the *K*_m_ values for ascorbate, being 140–180 μM for the PHDs and 260 μM for FIH (12). Normally, adequate levels are maintained by active transport from the plasma and most tissues achieve low millimolar intracellular concentrations (19, 20). However, if supply is limited either by low plasma levels or due to poor vascular delivery, tissue ascorbate levels can drop dramatically (21). Therefore, in order to maintain ascorbate saturation and optimal HIF-hydroxylase activity, cells require good accessibility and adequate circulating levels, both of which may be compromised in patients with solid tumors (22, 23).

Hence we propose that if tumor ascorbate is limiting, the HIF hydroxylases could be inhibited and the hypoxic response exacerbated. Improved supply of ascorbate could therefore provide an anti-cancer mechanism by optimizing HIF-hydroxylase activity and down-regulating HIF-1. There is some evidence that this can occur. In mouse models, ascorbate supplementation reduces tumor growth in a HIF-1-dependent manner (24, 25). We also reported an association between low ascorbate levels and high HIF-1 activation in human endometrial tumors (26), the first demonstration of an association between ascorbate and HIF-1 in a human tumor. We now report the analysis of surgically removed human colorectal cancer tissue.

Colorectal cancer is prevalent worldwide, with a high mortality rate (27) and is known to have increased HIF-1 expression that is associated with poor outcome (3, 28, 29). We have measured ascorbate levels, HIF-1α protein levels, and three HIF-1-controlled pro-survival target gene products; Bcl-2/adenovirus E1B 19 kDa interacting protein 3 (BNIP3), glucose transporter 1 (GLUT-1), and vascular endothelial cell growth factor (VEGF), in tumor and patient-matched adjacent normal tissue and compared these results to clinico-pathological data. We have complete 6-year follow-up data for this cohort of patients and have analyzed the impact of tumor ascorbate and HIF-1 activation on disease-free and overall survival.

## Materials and Methods

### Human ethics, colorectal cancer patients, and follow-up

Colorectal tumor and paired normal tissue samples (*n* = 49 patients with *n* = 50 pairs of samples) were obtained from the Cancer Society Tissue Bank, Christchurch (CSTBC). The samples were selected at random to represent a spread of male and female patients (42 and 58%, respectively), with a variety of tumor grades, stages, and sizes (Table 1). Donors gave written consent allowing research on their samples and ethical approval was granted by the Upper South B Regional Ethics Committee, New Zealand.

**Table 1:** **Clinical features of the colorectal cancer tissue sample set**.

Clinical parameter	*n* (%)
**GRADE**	
1 (Well differentiated)	9 (18)
2 (Moderately differentiated)	20 (40)
3 (Poorly differentiated)	21 (42)
**AJCC STAGE**	
1	7 (14)
2A	12 (24)
2B	6 (12)
3A	1 (2)
3B	11 (22)
3C	10 (20)
4	3 (6)
**SPECIMEN POSITION**	
Right ascending colon	17 (34)
Left descending colon	5 (10)
Transverse colon	1 (2)
Cecum	3 (6)
Sigmoid colon	9 (18)
Rectum	1 (2)
ND	14 (28)
**TUMOR SIZE**	
≤50 mm	32 (64)
>50 mm	17 (34)
ND	1 (2)
**NECROSIS**	
No	23 (46)
Yes	7 (14)
ND	20 (40)
**LYMPH/VASCULAR INVASION**	
No	32 (64)
Yes	17 (34)
ND	1 (2)

*AJCC, American Joint Committee on Cancer; ND, no data*.

Colorectal cancer patients who had gifted tissue between September 1998 and July 2008 were investigated. All colorectal patients treated at Christchurch hospital are offered standard care: post-operative adjuvant chemotherapy (intravenous weekly 5-fluorouracil with leucovorin, capecitabine, or an oxaliplatin combination) for patients with nodes involved and also node-negative patients with adverse features. Patients are followed up with 6-monthly clinical assessment and blood carcinoembryonic antigen, with an annual and then 3-yearly colonoscopy. Follow-up was documented until December 2012. Stage 4 patients with metastasis on presentation were excluded (*n* = 3), as their disease was considered as already progressed, and another patient (*n* = 1) was excluded as metastasis was discovered during surgery. In addition, patients who did not recover from surgery (i.e., died within 10 days of surgery) were excluded (*n* = 2). Two tumor samples were obtained from a single patient who presented with two independent primary tumors 4 years apart – survival data was applied to the first tumor surgery only, leaving 43 patients to be included in survival analysis.

### Tissue preparation

All colorectal tumor tissues were obtained as part of surgical resection with both tumor and normal tissue (from uninvolved mucosa ≥100 mm from the tumor edge) collected. Tissue samples were flash-frozen in liquid N_2_ within an hour of collection and stored at −80°C. Frozen samples were ground to a fine powder in liquid nitrogen, the collected tissue weighed, and 10 mM phosphate buffer (pH 7.4) added to make a homogenous suspension that was used to measure DNA (cellularity), tissue ascorbate content, and HIF-1α protein and gene product expression (26).

Hypoxia-inducible factor-1α was detected with mouse anti-human HIF-1α (1:1,000) (BD Biosciences, San Jose, CA, USA); BNIP3 with goat anti-human BNIP3 (1:1,000) (R&D Systems, Minneapolis, MN, USA); and GLUT-1 with rabbit anti-human GLUT-1 (1:3,000) (Abcam, New Zealand). Anti-β-actin primary antibody (1:10,000), horseradish peroxidase (HRP)-conjugated goat anti-mouse (1:2,500), rabbit anti-goat (1:20,000), and goat anti-rabbit (1:20,0000) were from Sigma Aldrich, St Louis, MO, USA. Protein detection was with ECL™ Plus detection reagent (GE Healthcare/Amersham Biosciences, Buckinghamshire, UK). Human VEGF protein was measured using a DuoSet ELISA from R&D Systems. Complete Mini protease inhibitor cocktail was from Roche Diagnostics (Mannheim, Germany).

### DNA content

Cell content was detected by monitoring DNA content of the tissue homogenate, diluted 1:25 and disrupted in 10 mM phosphate buffer, pH 7.4. Propidium iodide was added (1 mg/ml; 10 min, RT) and fluorescence measured at 544–590 nm, relative to a standard curve of purified calf thymus DNA.

### Ascorbate measurement

Ascorbate was extracted from the tissue homogenate by immediately adding a 1:1 volume of 0.54 M perchloric acid containing 50 mM diethylenetriaminepentaacetic acid (DTPA). The protein was pelleted and the supernatant analyzed by reverse-phase high performance liquid chromatography with electro-chemical detection (HPLC–ECD; mobile phase: 80 mM sodium acetate, pH 4.8, with 0.54 mM DTPA as previously described) (30). A fresh standard curve of sodium-l-ascorbate was prepared for each run.

### Western blotting

Hypoxia-inducible factor-1α, GLUT-1, BNIP3, and β-actin protein levels were measured by Western blotting. Homogenates were standardized to 15 μg DNA per 100 μl sample buffer (60 mM Tris pH 4.8, 2% SDS, 20% glycerol, 0.1 M DTT, Complete Mini protease inhibitor cocktail, and bromophenol blue) (26). After detection of the primary target, the membranes were re-probed for β-actin as a loading control. Images were captured using a ChemiDoc XRS gel documentation system (Bio-Rad Laboratories, Hercules, CA, USA) and densitometry performed using Quantity One^®^ software (Bio-Rad). Each protein was quantified relative to β-actin, and to normalize the signal between blots, quantified relative to a positive control for each protein of previously analyzed endometrial tumor tissue total protein (26).

### VEGF ELISA

Vascular endothelial cell growth factor protein levels were measured in the tissue homogenate, diluted 1:25 in 10 mM phosphate buffer, pH 7.4, by a commercial ELISA (R&D Systems) according to the manufacturer’s instructions.

### Statistical analyses

Data were analyzed using SPSS 19.0 with the alpha level set to 0.05. The Kolmogorov–Smirnov test determined the distribution of each variable. All data was non-parametric except for the ascorbate levels. For differences between normal and tumor data, the Mann–Whitney test was used and for differences between tumor grades, the Wilcoxon signed ranks test was used. Correlations were determined using Spearman’s correlation coefficient. Linear regression analysis was also used in tumor size and HIF-1 pathway score analyses. The Student’s *t*-test determined differences in ascorbate levels. Survival analyses were performed in R 2.14 (Vienna, Austria).

## Results

### HIF-1α and target gene protein levels

**Figure 1:**
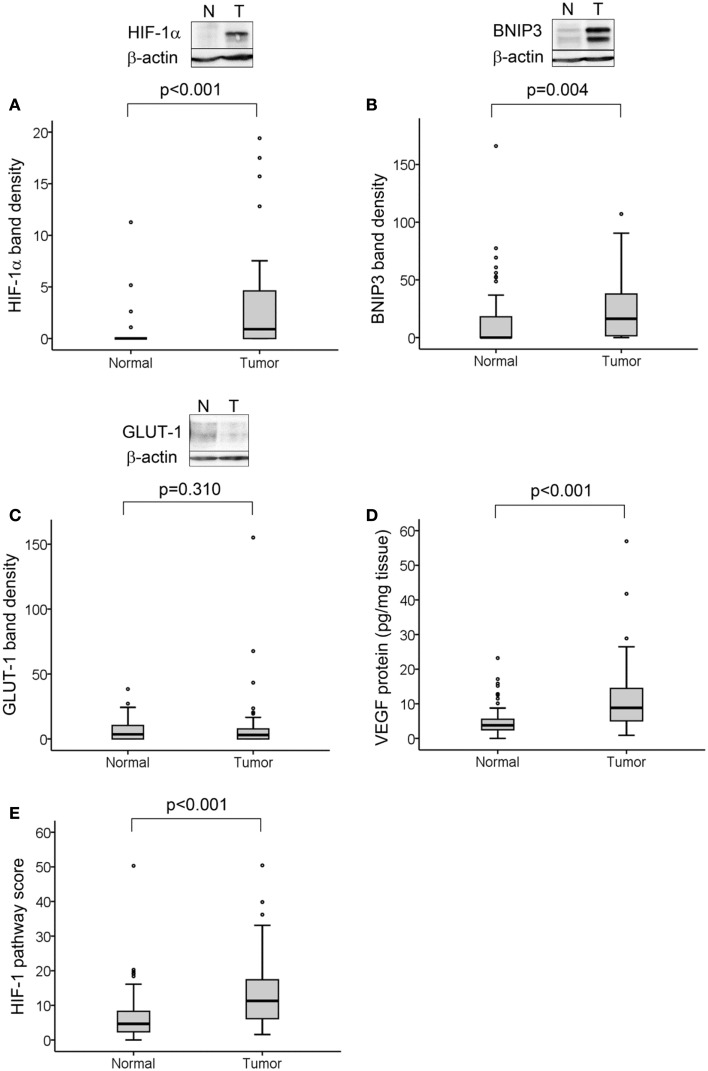
**HIF-1 activation in normal and tumor colorectal tissue samples**. Representative Western blots from normal and tumor tissue samples are shown for HIF-1α **(A)**, BNIP3 **(B)**, and GLUT-1 **(C)**, together with boxplots of densitometry results from all samples normalized to the same positive control for each protein and to β-actin loading control. VEGF protein levels were measured by ELISA **(D)**. All proteins except GLUT-1 were elevated in tumor compared to normal tissue. HIF-1 pathway score **(E)**, was determined for each sample from the combined analysis of all four protein levels, normalized as percent maximum expression and the mean taken. This measure was significantly higher in tumor compared to normal tissue. For all measures, *n* = 50 cases with normal and tumor tissue samples for each; significance determined by Mann–Whitney test.

Hypoxia-inducible factor-1α protein was detected in 29 of 50 tumors (58%) and in 4 of 50 normal tissue samples (8%). We determined significantly higher levels of HIF-1α protein in tumor compared to normal tissue (*p* < 0.001; Figure 1A). BNIP3 was detected in 38 of 50 tumors (76%) and 21 of 50 normal tissues (42%) and was higher in tumor compared to normal tissue (*p* = 0.004; Figure 1B). GLUT-1 was detected in 36 of 50 tumors (72%) and 34 of 50 normal tissues (68%) with no difference in levels between normal and tumor samples (Figure 1C). VEGF protein was detectable in 50 of 50 tumors (100%) and 49 of 50 normal tissues (98%) with higher levels in tumor than normal tissue (*p* < 0.001; Figure 1D).

As ascorbate can affect HIF-1α protein levels and transcriptional activity, the values for HIF-1α protein and expression of BNIP3, GLUT-1, and VEGF were combined to assess the activation of the HIF-1 pathway in relation to ascorbate content. The level of each protein was normalized relative to the maximum expression and the mean of all four values for each sample was assigned as the “HIF-1 pathway score” (26). This was verified by correlation with the individual protein levels (Table 2). The HIF-1 pathway score was significantly higher in tumor compared to normal tissue (Figure 1E; *p* < 0.001).

**Table 2:** **Spearman’s correlations between colorectal tumor tissue ascorbate content, HIF-1 activation markers, and tumor size**.

		Ascorbate content (nmol/μg DNA)	Tumor: normal ascorbate ratio	HIF-1α band density	GLUT-1 band density	BNIP3 band density	VEGF (pg/mg tissue)	HIF-1 pathway score	Tumor size (mm)
Ascorbate content (nmol/μg DNA)	*r*		**0.651**^a^	0.006	−0.223	−**0.302**^b^	−**0.321**^b^	−**0.445**^a^	−0.256
	*p*		**0.000**	0.970	0.120	**0.033**	**0.023**	**0.001**	0.076
Tumor:normal ascorbate ratio	*r*	**0.651**^a^		0.112	−**0.357**^b^	−0.134	−**0.305**^b^	−**0.364**^a^	−0.112
	*p*	**0.000**		0.437	**0.011**	0.354	**0.031**	**0.009**	0.445
HIF-1α band density	*r*	0.006	0.112		0.026	0.135	0.153	**0.284**^b^	0.127
	*p*	0.970	0.437		0.856	0.351	0.288	**0.046**	0.385
GLUT-1 band density	*r*	−0.223	−**0.357**^b^	0.026		0.058	0.012	**0.284**^b^	−0.069
	*p*	0.120	**0.011**	0.856		0.691	0.932	**0.045**	0.637
BNIP3 band density	*r*	−**0.302**^b^	−0.134	0.135	0.058		0.124	**0.781**^a^	0.083
	*p*	**0.033**	0.354	0.351	0.691		0.393	**0.000**	0.572
VEGF (pg/mg tissue)	*r*	−**0.321**^b^	−**0.305**^b^	0.153	0.012	0.124		**0.493**^a^	0.172
	*p*	**0.023**	**0.031**	0.288	0.932	0.393		**0.000**	0.236
HIF-1 pathway score	*r*	−**0.445**^a^	**0.364**^a^	**0.284**^b^	**0.284**^b^	**0.781**^a^	**0.493**^a^		0.057
	*p*	**0.001**	**0.009**	**0.046**	**0.045**	**0.000**	**0.000**		0.695
Tumor size (mm)	*r*	−0.256	−0.112	0.127	−0.069	0.083	0.172	0.057	
	*p*	0.076	0.445	0.385	0.637	0.572	0.236	0.695	

*For all analyses *n* = 50, except for tumor size, where *n* = 49. Significant associations are shown in bold font*.

*^a^Correlation is significant at the 0.01 level (two-tailed)*.

*^b^Correlation is significant at the 0.05 level (two-tailed)*.

### Ascorbate content in tumor and normal tissue samples

**Figure 2:**
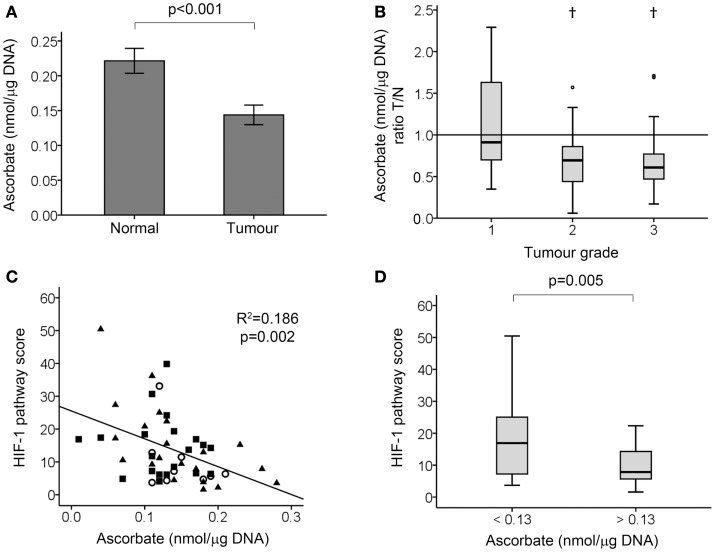
**Tumor ascorbate analysis and HIF-1 pathway activation in colorectal tumor samples**. **(A)** Tissue ascorbate levels are significantly lower in tumour compared to normal tissue samples as measured by paired student’s *t*-test (*n* = 50 normal and tumour samples, data represent mean ± SEM). **(B)** Tumour: normal tissue ascorbate ratio by tumour grade. Boxplot of the ratio of ascorbate in tumour relative to matching normal tissue, showing high grade tumours have significantly less ascorbate than surrounding normal tissue from the same patient. †*p* < 0.01, normal vs. tumour by Wilcoxon signed ranks test; *n* = 9, 20, and 21 for grade 1, 2, and 3, respectively. **(C)** Scatterplot showing tumor tissue ascorbate levels are inversely related to the HIF-1 pathway score (linear regression analysis). Grade 1, ◯ (*n* = 9), grade 2, ■ (*n* = 20), and grade 3, ▲ (*n* = 21). **(D)** Boxplot showing division of tumor samples according to the median ascorbate level, with ascorbate-deficient tumors (ascorbate <0.13 nmol/μg DNA; *n* = 25) having significantly higher HIF-1 pathway scores than ascorbate-replete tumors (ascorbate >0.13 nmol/μg DNA; *n* = 25) as determined by Mann–Whitney test.

Tissue ascorbate content was measured using HPLC–ECD (Figure 2). The stability of ascorbate in banked tissue as well as our protocol for ascorbate extraction has previously been verified, with no loss from intact tissue at room temperature for up to 5 h, nor upon long-term storage at −80°C (26). Tumor tissue had increased DNA content compared with normal tissues (4.50 ± 1.49 and 2.26 ± 1.04 μg DNA per milligram wet weight ± SD, respectively; *p* < 0.001), consistent with increased cell density of tumor tissue. As it is the intracellular level of ascorbate that is relevant to the control of HIF-1, the ascorbate measurements were standardized against the DNA content and expressed as nanomoles ascorbate per microgram DNA. There was a strong correlation between DNA content and absolute ascorbate levels (mg per 100 g wet weight) in both normal and tumor tissues (Spearman’s rho = 0.547 and 0.482, respectively; *p* < 0.001), suggesting that most of the ascorbate in the tissues was indeed intracellular. Furthermore, the DNA content did not increase with tumor grade, indicating that aneuploidy is unlikely to confound the measurement.

Two parameters were measured; (1) absolute ascorbate measurements in the tumor tissue, that reflect plasma supply, vascular delivery, and cellular uptake, and (2) the ability of the tumor to accumulate ascorbate relative to the surrounding tissue (shown as a ratio of tumor: normal tissue), which allows for correction for the individual patient’s ascorbate status and most likely reflects vascular delivery.

When measuring the absolute tumor ascorbate levels, significantly lower levels were found to be present than in normal tissue (*p* < 0.001; Figure 2A). When expressed relative to normal tissue, the median tumor ascorbate ratio was 0.70 (*p* = 0.001). This difference was attributable to the high grade tumors with the median tumor: normal ascorbate ratios being 0.910, 0.695, and 0.610 for grade 1, 2, and 3 tumors, respectively (Figure 2B). This difference was not due to variations in the cellularity of the three tumor grades, as each had the same DNA content [4.95 ± 1.50, 4.14 ± 1.56, and 4.65 ± 1.40 μg DNA per milligram wet weight (means ± SD), for grades 1, 2, and 3, respectively]. These results indicate that high grade colorectal tumor tissue has a reduced capacity to accumulate ascorbate compared to surrounding normal tissue, and also compared to low grade tumor tissue.

### Ascorbate and HIF-1 activation markers

Tumor ascorbate content and the HIF-1 pathway score were evenly distributed for all tumor grades (Figure 2C), with samples with high and low absolute ascorbate content in each grade. Analysis of the data showed a strong inverse relationship between the HIF-1 pathway score and tumor ascorbate content (*r* = −0.445, *p* = 0.001; Table 2; Figure 2C). VEGF and BNIP3 protein levels were also inversely correlated to tumor ascorbate content (Table 2). The ascorbate ratio (tumor:normal) was inversely correlated to VEGF and GLUT-1 protein levels, as well as to the overall HIF-1 pathway score (Table 2).

When samples were separated according to the median ascorbate content (0.13 nmol/μg DNA), the ascorbate-replete tumors had significantly lower HIF-1 pathway scores than low ascorbate tumors (Figure 2D; *p* = 0.005).

**Figure 3:**
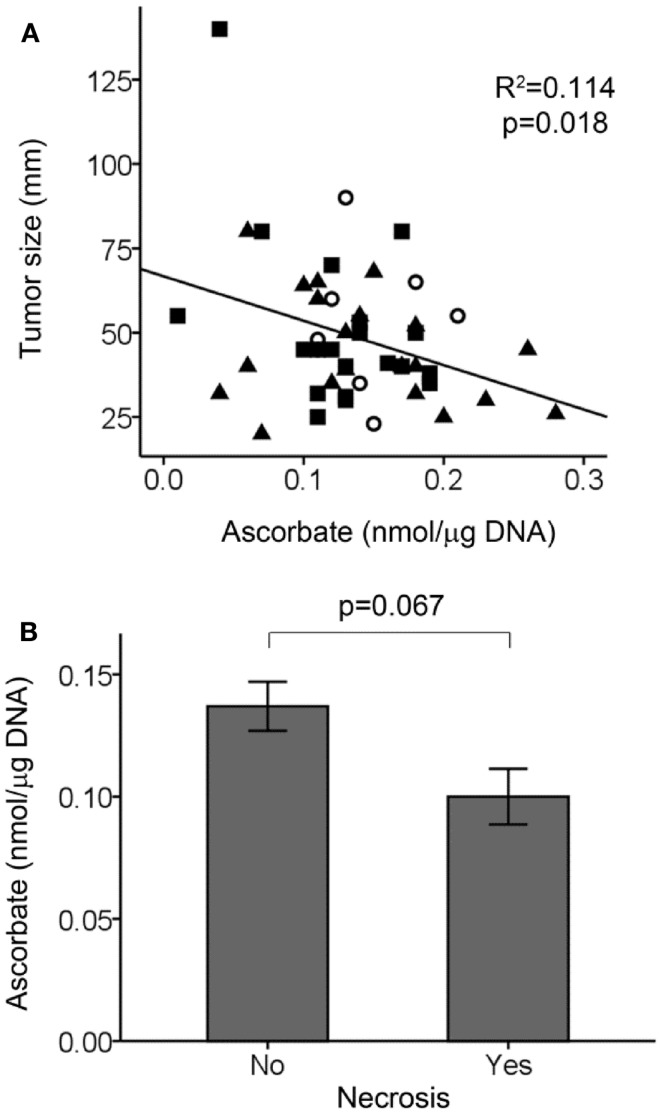
**Tumor ascorbate levels in relation to tumor size and necrosis**. **(A)** Scatterplot showing an inverse relationship between tumor ascorbate levels and tumor size (linear regression analysis). Grade 1, ◯ (*n* = 9), grade 2, ■ (*n* = 20), and grade 3, ▲ (*n* = 21). **(B)** Tumors positive for necrosis (*n* = 7) tended to have lower ascorbate levels than tumors with no detectable necrosis (*n* = 23) as determined by unpaired Student’s *t*-test. Data represent mean ± SEM.

There was an association between tumor size and ascorbate content, with larger tumors having less ascorbate (Figure 3A). In contrast, there was no association between normal tissue ascorbate levels and tumor size (*r* = 0.024, *p* = 0.292). We did not find any associations between tumor ascorbate levels and the HIF-1 pathways score with AJCC stage (*p* = 0.781) or lymph/vascular invasion status (*p* = 0.817) of the tumors. There was a tendency for tumors positive for necrosis to have less ascorbate, with data available for 30 out of 50 tumors (Figure 3B; *p* = 0.067).

### Colorectal cancer patient survival

We were able to map 6-year post-surgery survival data for 43 of the 49 patients. Excluded from the analysis were four patients who had metastatic disease at time of surgery and to whom the disease-free status does not apply, and two patients who died of complications within days of surgery. The cohort was divided into “high” (*n* = 22) and “low” (*n* = 21) categories based on the median tissue ascorbate content or median tumor:normal ratio.

**Figure 4:**
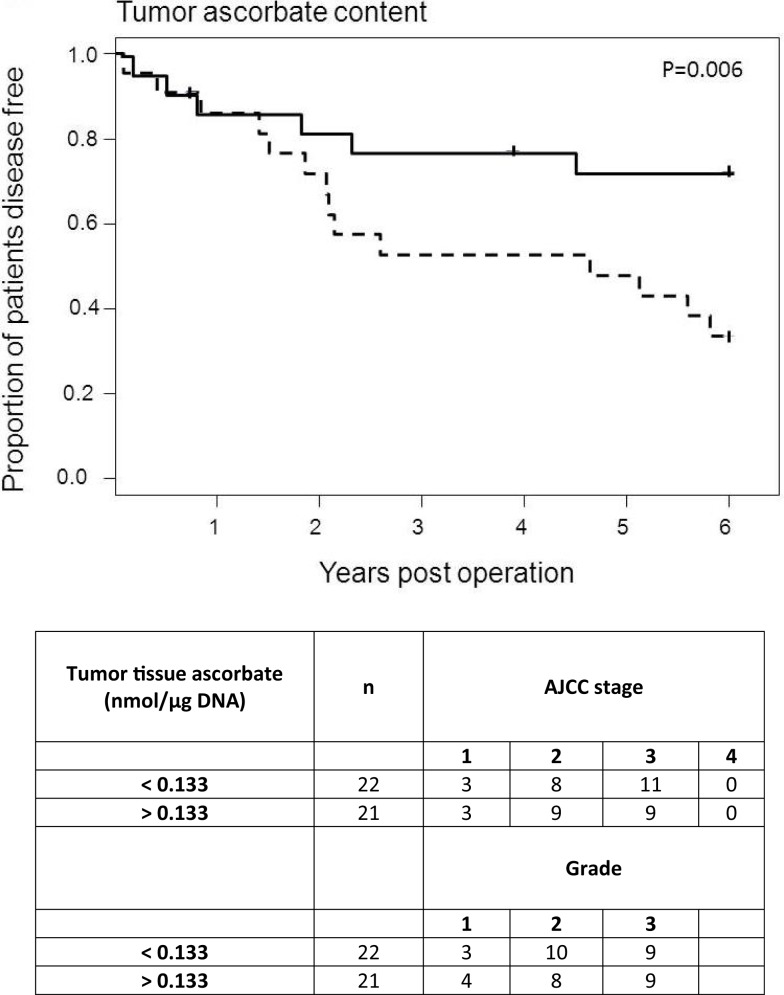
**Disease-free survival of colorectal cancer patients according to ascorbate**. Each tumor ascorbate parameter was divided into “high” (*n* = 22, solid lines) and “low” (*n* = 21, dotted lines) categories based on the median value. The proportion of disease-free patients was plotted over time since their tumor resection surgery. Each step down on the figures represents a patient whose cancer has progressed, and each tick mark represents cessation of follow-up of a patient. Low tumor tissue ascorbate content is significantly associated with shorter disease-free survival (*p* = 0.006; median = 0.133 nmol/μg DNA).

The results indicate a distinct disease-free survival advantage for those patients with higher tumor ascorbate levels (*p* = 0.006; Figure 4). Although the sample size was too small to include covariates in the analysis, the “high” and “low” groups contained similar proportions of tumor grades and stages, indicating that there is no bias by disease severity (Figure 4). In contrast with this, the ascorbate content of normal tissue was not related to patient outcome (*p* = 0.614). The results were similar whether ascorbate content was measured as milligram per 100 g of tissue or nanomole per microgram DNA (Table 3). In addition, inclusion of the six excluded patients did not change the interpretation of the results with similar findings observed when analyses were carried out on *n* = 49 (Table 3).

**Table 3:** **Patient 6-year disease-free survival percentages according to tumor and normal tissue ascorbate measurements**.

	Median value	<Median (%)	>Median (%)	95% CI		*p*
**ASCORBATE AS nmol/μg DNA (*n* = 43)**						
Tumor tissue	0.133	33.5	72.4	11.3	66.6	0.006*
Normal tissue	0.205	57.3	49.6	−37.5	22.2	0.614
Tumor:normal ratio	0.709	39.7	67.0	−1.6	56.3	0.064
**ASCORBATE AS nmol/μg DNA (*n* = 49)**						
Tumor tissue	0.132	30.8	63.8	6.2	59.7	0.016*
Normal tissue	0.212	56.7	39.6	−45.1	10.8	0.230
Tumor: normal ratio	0.695	29.2	66.6	11.1	63.7	0.005*
**ASCORBATE AS mg/100 g TISSUE (*n* = 43)**						
Tumor tissue	12.2	38.3	67.9	1.0	58.2	0.043*
Normal tissue	8.0	46.5	59.1	−17.4	42.5	0.410
Tumor: normal ratio	1.50	40.9	66.5	−3.3	54.5	0.083
**ASCORBATE AS mg/100 g TISSUE (*n* = 49)**						
Tumor tissue	11.9	35.2	59.5	−3.3	51.9	0.084
Normal tissue	8.2	46.9	48.0	−27.4	29.7	0.938
Tumor: normal ratio	1.46	29.2	66.3	10.8	63.6	0.006*

Estimates from log-rank tests (dichotomous at the median level); *p*, significance (*). Bold font indicates a significant association.

Patients with high ascorbate in their tumors relative to normal tissue had between 141 and 1,094 additional disease-free days in the first 6 years after surgery (*p* = 0.011), with the range likely reflecting the small sample size (*n* = 43). Assessment of patient overall survival did not show any associations or trends with respect to ascorbate: this is likely to be due to median survival not being reached, to the relatively advanced average age (78 years) and because we did not have cause of death information and could not exclude other confounding factors.

## Discussion

Whether ascorbate is of benefit to cancer patients has long been argued, with little information being available to substantiate claims of efficacy. Recent information from both human and animal studies is highly suggestive of a clinical benefit, and several mechanisms of action have been proposed (31–33). To our knowledge, our retrospective analysis of colorectal cancer is the first study to measure patient outcome in relation to tumor ascorbate content and to show that this is associated with activation of HIF-1, a known driver of tumor growth and metastasis. We found that the ascorbate content of tumor samples is associated with HIF-1 activation, tumor characteristics, and metastases. Disease-free survival was associated with the ascorbate level in the tumor, but not in normal tissue, suggesting that the most important determinant is the amount of ascorbate in the tumor and not the patient’s overall ascorbate status.

We previously showed that decreased ascorbate levels are associated with high HIF-1 activation in endometrial cancer (26), but patient survival data was not available. The current study has corroborated our previous findings in endometrial cancer, making an important advance in determining whether sub-optimal tumor ascorbate leads to over-activation of HIF-1. It has also indicated that ascorbate levels influence tumor progression.

Our findings are supported by *in vitro* studies showing that ascorbate supplementation can inhibit HIF-1 activation in cancer or primary cells (16, 17, 34, 35). Furthermore, *in vivo* observations showed that increased ascorbate reduced HIF-1-dependent tumor genesis in wild-type mice (25). Enhanced tumor growth and metastasis has been also shown in *Gulo^−/−^* ascorbate-deficient mice compared to ascorbate-supplemented mice (36, 37), and this was associated with increased HIF-1 target gene expression (MMP9 and VEGF) (37).

Our previous study with endometrial cancer samples was the first measurement of ascorbate levels and HIF-1 activation in human cancer. The current study, with the acquisition of patient survival data, is consistent with the hypothesis that low tumor ascorbate content may lead to HIF-1-dependent tumor progression.

The use of ascorbate to treat cancer is contentious, with little clinical evidence to support the practice. Despite this, the administration of high-dose intravenous ascorbate to cancer patients is widespread (38). Pharmacokinetic studies have demonstrated more than 100-fold higher plasma ascorbate levels after intravenous than after oral administration (39). This increased availability may explain some of the discrepancies between the definitive clinical trial in the 1980s (40) using oral ascorbate to treat cancer and earlier studies by Cameron and Pauling using both oral and intravenous ascorbate (41, 42). Because of new understanding of the pharmacokinetics, possible roles for ascorbate in cancer are now being investigated, spurring the growing body of pre-clinical evidence demonstrating the effectiveness of ascorbate against tumor cell growth and survival (16, 25, 31, 33, 43–46).

We found that tumor tissue contained up to 40% less ascorbate than adjacent normal tissue. The reasons for this are unclear; however it is known that accumulation of intracellular ascorbate is influenced by expression and activity of the active transporter SVCT2 (47) and availability of ascorbate in the extracellular milieu. The latter largely reflects the delivery of ascorbate from plasma to the tissues and this depends on both the plasma levels as well as an effective vascular network. These can both be affected in cancer patients who reportedly have decreased circulating ascorbate (22, 23) and whose tumors have dysfunctional vasculature (48). Poorly functioning tumor vasculature results in regions of hypoxia and it is highly plausible that this would also compromise ascorbate supply to tumor cells and also to the many other cell types (such as endothelial cells, fibroblasts, and immune cells) that make up the complex tumor microenvironment.

Our results indicate that it is the absolute amount of ascorbate in the tumor tissue (and which was independent of grade and stage), rather than the patient’s overall ascorbate status, that was associated with disease-free survival. This suggests that increasing tumor tissue ascorbate levels may be beneficial.

Delivery of ascorbate to tumor tissue may be a limiting factor for its therapeutic value. It is unknown what plasma concentrations are required for adequate tumor tissue penetration and whether this can be achieved through dietary intake or whether intravenous administration may be necessary. Maximum plasma ascorbate levels reach only ~100 μM through gastrointestinal absorption, whereas intravenous administration can result in plasma concentrations above 10 mM (39). Pharmacokinetic data on ascorbate in tumor tissues following administration would be particularly valuable in order to determine the optimal dose regimen to achieve cellular levels optimal for HIF-hydroxylase activity (~1–3 mM) (14).

Hypoxia-inducible factor-1 is particularly relevant to colorectal cancer, with HIF-1α expression being identified as an independent indicator of poor prognosis in a large prospective study of 731 patients (3). In addition, whereas HIF-1α alone was not significant, in combination with two of its target gene products (CXCR4 and VEGF) it was associated with distant metastasis and poor disease-free survival (49). In another study, high VEGF expression was also found to be associated with poor survival, whereas HIF-1α was borderline significant (50). These studies also highlight the dual control of the HIF-1 transcriptional response by the PHDs and FIH and the importance of measuring HIF-1α protein levels and its downstream gene products in combination, as we have done here. That both VEGF and BNIP3, but not HIF-1α, protein levels were inversely correlated to the tumor ascorbate content agrees with the observation that FIH may be more dependent on ascorbate for activity than the PHDs (12). Thus, ascorbate may have a greater influence on curbing HIF-1 transcriptional activity than it does on protein stabilization of HIF-1α.

If ascorbate is to be considered as a potential cancer therapy, there needs to be a clear understanding of its pharmacokinetics and mechanism of action in tumor tissue before appropriately designed clinical trials begin. Ascorbate may be active only in particular tumor types that are HIF-dependent or have a supportive microenvironment. Moreover, the dosing regimen needs to be clarified in order to achieve effective tumor tissue ascorbate levels. As there is considerable skepticism regarding the usefulness of ascorbate in treating cancer, addressing these important mechanistic issues will shed light on previous clinical research and direct the nature of future human studies.

## Author Contributions

Caroline Kuiper performed the analyses, was responsible for study design and writing the manuscript. Gabi U. Dachs was responsible for study design and retrieval of survival data. Delwyn Munn was responsible for retrieval of survival data. Margaret J. Currie was involved in study design, retrieval of survival data, and writing the manuscript. Bridget A. Robinson is the Clinical Oncologist with oversight of patient data and was involved in study design and writing the manuscript. John F. Pearson is a biostatistician and carried out some of the statistical analyses. Margreet C.M. Vissers was responsible for study design, overall supervision of the project, and writing the manuscript.

## Conflict of Interest Statement

The authors declare that the research was conducted in the absence of any commercial or financial relationships that could be construed as a potential conflict of interest.
